# The association between problematic smartphone use and the severity of temporomandibular disorders: A cross-sectional study

**DOI:** 10.3389/fpubh.2022.1042147

**Published:** 2022-12-23

**Authors:** Ya-Peng Pei, Han-Chao Li, Jia-Wei Zhong, Xin-Lin Gao, Chu-Qiao Xiao, Yuan Yue, Xin Xiong

**Affiliations:** ^1^Department of Orthodontics, National Clinical Research Center for Oral Diseases, State Key Laboratory of Oral Diseases, West China Hospital of Stomatology, Sichuan University, Chengdu, China; ^2^Department of Prosthodontics, National Clinical Research Center for Oral Diseases, State Key Laboratory of Oral Diseases, West China Hospital of Stomatology, Sichuan University, Chengdu, China

**Keywords:** temporomandibular disorders, problematic smartphone use, psychological problems, temporomandibular joints, young adult

## Abstract

**Objective:**

To evaluate the prevalence of different types of temporomandibular disorders (TMD) symptoms in young adults and determine their associations with problematic smartphone use (PSU).

**Methods:**

The data of the study were collected from local university students through an online questionnaire survey. Demographic information, Fonseca Anamnestic Index (FAI), Smartphone Addiction Scale-Short Version (SAS-SV), and Patient Health Questionnaire-4 (PHQ-4) responses were gathered electronically and analyzed using multiple logistic regression analysis.

**Results:**

There were 163 male and 307 female respondents were participated in this study. The prevalence of PSU and TMD were 83.6% and 66.4%, respectively. There was a moderate statistical correlation between PSU and TMD among young adults (*r* = 0.31, *p* < 0.01). The logistic regression model revealed that the risk of TMD was 1.77 times higher in people with PSU than in those without PSU (OR = 1.77; 95% CI 1.04–3.06). PSU is a risk factor for pain-related TMD (OR = 1.81; 95% CI 1.08–3.04) but not intra-articular TMD.

**Conclusion:**

Subjects showed high prevalence of both TMD and PSU. People with PSU experienced more severe and frequent pain-related rather than intra-articular TMD symptoms than those without PSU. By reducing the problematic smartphone use, the risk factor of TMD might be avoided.

## Introduction

Smartphones have become indispensable for people and influence people's lifestyles. According to the 49th China statistical report on Internet development, as of December 2021, the number of mobile internet users in China was 1.03 billion, accounting for 99.7% of the total number of Chinese Internet users (1). The wide use of smartphones brings convenience as well as health hazards. One of the most prominent problems is problematic smartphone use (PSU). The overuse of smartphones had been proven to be one of the major contributing factors of many physical symptoms, including discomfort and pain in neck, eyes, shoulder, and wrists (2–5). The overuse of smartphones had also been associated with many psychological conditions, such as depression, anxiety, and low self-esteem (6–8).

Temporomandibular disorders (TMDs) are a set of more than 30 health disorders associated with temporomandibular joints (TMJ) and the muscles and tissues of the jaw (9). The prevalence of TMDs is 30% from Chinese population, even 60% from young adults (10, 11). TMD seriously affects people's quality of life, resulting in physical pain, psychological problems, bad life performance and large treatment cost. Besides, chronic TMD patients exhibited greater psychological distress, such as somatization symptoms, stress, and anxiety (12). The most common symptoms of TMDs include limited mouth opening, neck pain, TMJ pain and noise, and headache. The etiology of TMD is complex, which includes age, sex, parafunctional habits, and psychological problems (13, 14). In recent years, the prevalence of TMD has been increasing (15), which might be associated with the change of lifestyle. The Diagnostic Criteria for TMDs (DC/TMD) is the current benchmark for TMD diagnoses. According to the DC/TMD, TMD conditions could be categorized into pain-related (PT) and intra-articular (IT) joint disorders (16).

Although there is a lack of studies directly pointing out the relationship between TMD and PSU, some studies have shown that there may be a potential relationship between them. Researches have shown that people with smartphone overuse were more likely to present neck pain than those without smartphone overuse (17). A study has clarified that smartphone use can lead to poor head posture, such as severe head flexion (18). In this poor position, TMJ will suffer from adverse biomechanical effects resulting in TMD (19). And the head flexion angle while using smartphones is significantly larger than that while doing other activities, which may contribute to the occurrence of neck pain among people who are addicted to smartphones. Neck pain is one of the most common symptoms of TMD, which suggests it is worthy to study the association between PSU and TMD. In addition to possible biological agents, psychological problems are also common among people with PSU and TMD. Psychological problems such as depression and anxiety are one of the consequences of PSU (6), which are also risk factors for TMD (13). Therefore, a direct study of the relationship between PSU and TMD is required.

Considering the scarcity of research on this topic, this research aimed to research the relationship between specific TMD and PSU. The null hypothesis was followed: (a) there is no association between the prevalence of TMD and PSU; (b) there is no correlation between PT expression and PSU; (c) there is no correlation between IT expression and PSU.

## Materials and methods

### Design and sample

This cross-sectional study was approved by Ethics Committee of West China School of Stomatology of Sichuan University (Ethics number: WCHSIRB-CT-2022-240) and was conducted in conformity to the Declaration of Helsinki. All adult participants themselves provided informed consent. The study followed the Strengthening the Reporting of Observational Studies in Epidemiology (STROBE) recommendation (20).

**Figure 1:**
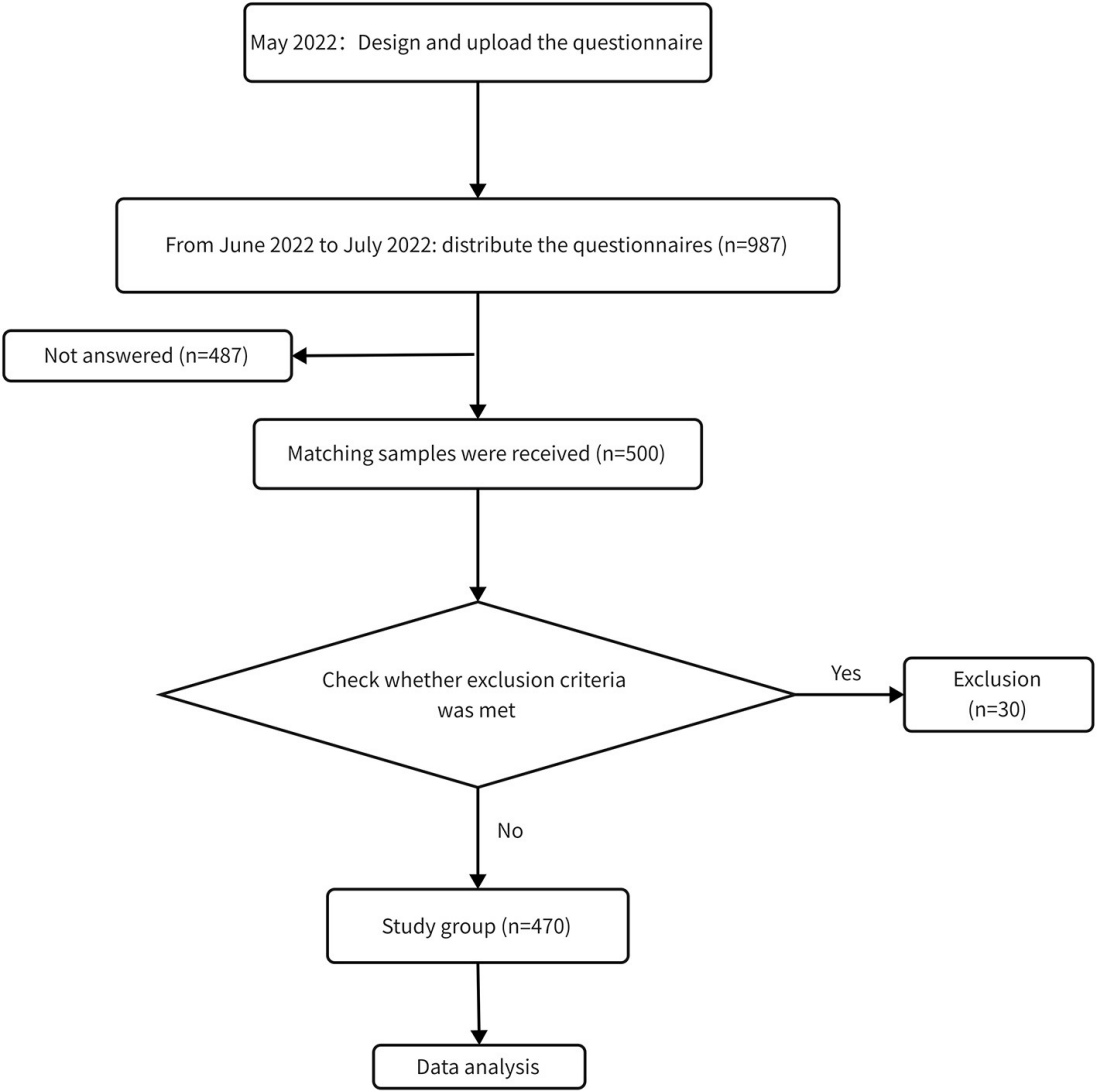
The flow chart of the sample inclusion.

Subjects who were older than 18 and offered informed consent were included in the study. Exclusion criteria: (a) prior orofacial trauma/surgeries, (b) having a diagnosis of systemic, metabolic neurological, or immune disorder or disease, (c) cognitive impairments and illiteracy, (d) all questionnaires were answered illogically, and (e) the questionnaire answering time was less. The questionnaire was uploaded to “Questionnaire Star,” the most widely used questionnaire website in China in May 2022. Then suitable subjects were recruited on campus from June 2022 to July 2022. Systematic random sampling was employed in selecting the subjects by student number. After that, we explained the purpose and content of the study to the subjects and sent a link to subjects after obtaining verbal informed consent. Subjects could complete the questionnaire by clicking the link (https://www.wjx.cn/vm/exIfAIw.aspx). The background and purpose of the survey were shown on the first page of the questionnaire. Each respondent contributed to the study without compensation and could leave an email for review. To reduce the bias, in the questionnaire design stage, the number of questions and the time to answer were controlled within a suitable range. The questionnaire had satisfactory validity and reliability. In total, we distributed questionnaires to 987 participants and received 500 matching samples. A total of 487 people refused to answer the questionnaire because they were not interested in it, did not have time or for privacy protection concerns. The response rate was 50.7%. A total of 30 invalid questionnaires were excluded, and 470 valid questionnaires were collected in the final analysis. A flow chart depicted the study (Figure 1).

### Measurement

The Smartphone Addiction Scale-Short Version (SAS-SV) developed by Kwon et al. (21) was widely used to evaluate smartphone addiction and had been proven to have good reliability and validity in the Chinese population by Luk et al. (22) in 2018. The scale comprises 10 items relating to the rate and duration of smartphone use, including “loss of control” (Items 1 and 8), “disruption” (items 2 and 10), “disregard” (items 3 and 7), “withdrawal” (items 4 and 5), “preoccupation” (item 6), and “tolerance” (item 9). Each item scores on a Likert scale of 1 (strongly disagree) to 6 (strongly agree). According to the threshold recommended for Chinese populations, total sum scores of ≥31 for males and ≥33 for females indicate PSU.

The prevalence and severity of TMD symptoms were measured with Fonseca Anamnestic Index (FAI), a self-assessment multi-dimensional instrument which includes ten items assessing pain and function-related TMD symptoms as well as TMD risk factors (23). Each item provides three response options: yes (10 points), sometimes (5 points) and no (0 points). Subjects with scores between 0 and 15 were considered to be TMD-free, while those with scores of 20 or more were considered to be TMD patients. And TMD patients can be categorized into mild TMD (20–40), moderate TMD (45–65) and severe TMD (70–100) (24). The short-form FAI (S-FAI) was employed to assess the presence of pain-related TMD and intra-articular TMD. The S-FAI consists of five TMD-specific items of the FAI (TMJ pain, masticatory muscle pain, TMJ sounds, opening, and jaw movement difficulties) (25). The sensitivity and specificity for Q3 and Q4 in relation to pain-related TMDs as well as for Q1, Q2, and Q5 in relation to intra-articular TMD were investigated. In this study, for Q3 and Q4 total sum scores of 5–20 denote the presence of pain-related TMD. For Q1, Q2 and Q5 total sum scores of 5–30 indicate the presence of intra-articular TMD.

The Patient Health Questionnaire-4 (PHQ-4) is an ultra-brief screening scale for depression and anxiety, which includes two separate scales, Patient Health Questionnaire-2 (PHQ-2) and Generalized Anxiety Disorder-2 (GAD-2). Individuals with a sum score of ≥3 for the PHQ-2 and GAD-2 were screened for depression and anxiety, respectively (26).

### Statistical analysis

The sample consisted of the college students in different majors. A minimum sample size of 327 was calculated by a sample size calculator (https://www.calculator.net/sample-size-calculator.html) based on a 95% confidence level, 5% sampling error, total students of 60,000, and a 31% prevalence of TMDs (27).

Statistical analyses were carried out with SPSS 25.0 version and EmpowerStats (http://www.empowerstats.com), X&Y Solutions, Inc. (Boston, MA). Statistical significance was accepted at the two-sided 0.05 level. Continuous variables were presented as the mean ± standard deviation (M ± SD), and categorical variables were presented as numbers with percentages. The Kolmogorov-Smirnov test was used to analyze the normality of the distribution of the data. Chi-square tests, ANOVA and *t* tests were conducted to compare groups. The Spearman correlation coefficient was used to analyze the association among FAI, SAS-SV, PHQ-2, and GAD-2 scores and the association between each item of FAI and PSU. When *r* = 0.1–0.3, 0.3–0.5 and 0.5–0.9, the correlation strength was classified as weak, moderate, and strong, respectively (28). Interaction and stratified analyses were conducted according to gender (male and female), income (< 3,000 and ≥3,000), depression and anxiety. Multiple logistic regressions were used to evaluate the associations between PSU and the risk of TMD. And the data were reported as odds ratios (ORs) and 95% confidence intervals (CI). Subjects without PSU were considered as the reference group and potential confounders were adjusted. Cronbach's α was used to measure the internal consistency of questionnaires. When Cronbach's α is 0.7–0.9, the scale is considered to be reliable.

## Results

### Demographic characteristics

A total of 470 participants were included in this study, including 163 males (34.7%) and 307 females (65.3%), the average age of which was 21.41 ± 2.91 year old. The overall prevalence of PSU was 83.6%. Females had a higher prevalence of PSU (85.67%) than males (79.75%). Meanwhile, no significant differences in PSU prevalence were observed among different income groups. Among people with depression, the prevalence of PSU (93.33%) was higher than that of people without depression (77.59%). And people with anxiety (94.19%) had a higher prevalence of PSU than people without anxiety (78.41%). The overall prevalence of TMD was 66.4%. Finally, the prevalence of PSU gradually increased in subgroups from no TMD (74.05%) to severe TMD (96.43%). The prevalence of PT and IT were 57.82% and 58.09%. Among people with PT (61.32%) and IT (61.07%), PSU prevalence was significantly higher than that of people without PT (38.68%) and IT (38.93%) (Table 1).

**Table 1:** Characteristics and PSU status of subjects (*N* = 470).

**Characteristics**		***N***	**Non-PSU**	**PSU**	***p***
Age (mean ± SD)		21.41 ± 2.91	21.11 ± 2.91	21.46 ± 2.91	0.14
Gender	Male	163	33 (20.25)	130 (79.75)	< 0.01
	Female	307	44 (14.33)	263 (85.67)	
Income	< 3,000	99	16 (16.16)	83 (83.84)	0.84
	≥3,000	371	61 (16.44)	310 (83.56)	
Depression	Yes	180	12 (6.67)	168 (93.33)	< 0.01
	No	290	65 (22.41)	225 (77.59)	
Anxiety	Yes	155	9 (5.81)	146 (94.19)	< 0.01
	No	315	68 (21.59)	247 (78.41)	
TMD	Severe	28	1 (3.57)	27 (96.43)	< 0.01
	Moderate	108	4 (3.70)	104 (96.30)	
	Mild	176	31 (17.61)	145 (82.38)	
	No	158	41 (25.95)	117 (74.05)	
PT	Yes	272	31 (40.26)	241 (61.32)	< 0.01
	No	198	46 (59.74)	152 (38.68)	
IT	Yes	273	33 (42.86)	240 (61.07)	< 0.01
	No	197	44 (57.14)	153 (38.93)	

SD, standard deviation; PSU, problematic smartphone use; TMD, temporomandibular disorder; PT, Pain-related temporomandibular disorder; IT, Intra-articular temporomandibular disorder.

**Figure 2:**
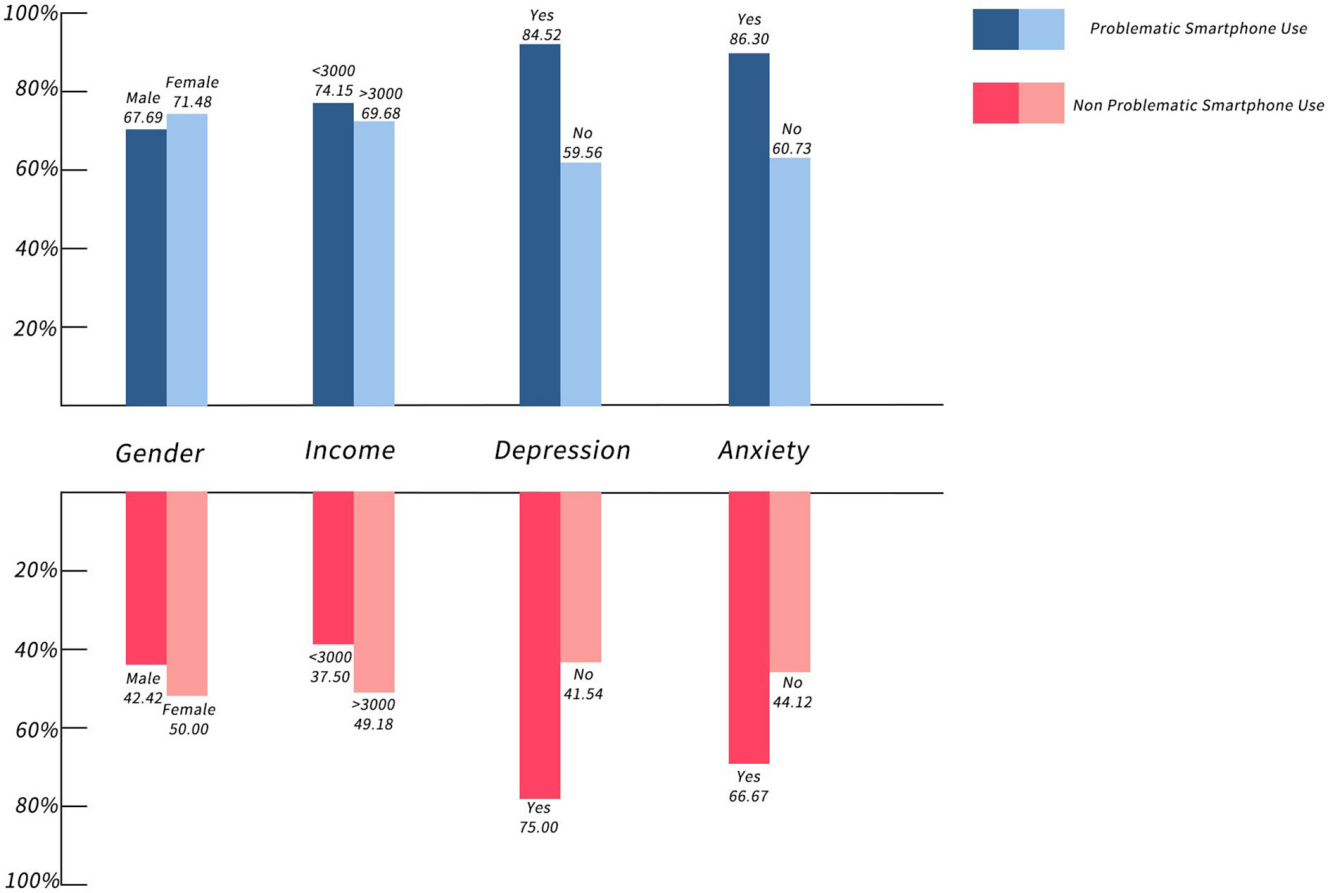
Prevalence of TMD in each subgroup of PSU and non-PSU.

The prevalence of TMD in females was higher than that in males. And, people with depression and anxiety had higher rates of TMD. Beside that, it is quite intuitive to see that the prevalence of TMD is higher in people with PSU than that in people without PSU in all subgroups (Figure 2).

### Association between PSU and TMD

In this study, Cronbach's α coefficients of SAS-SV, FAI and PHQ-4 were respectively 0.92, 0.83 and 0.85, which showed excellent internal reliability of these scales. The mean scores of SAS-SV and FAI were 40.82 and 30.14. Among students participating in this study, TMD had a moderate statistical correlation with PSU (*r* = 0.31, *p* < 0.01). Moreover, moderate significant correlations were also noted between TMD and depression (*r* = 0.39, *p* < 0.01) and anxiety (*r* = 0.41, *p* < 0.01) (Table 2).

**Table 2:** Spearman's correlation between the various questionnaires.

**Measurement**	**α**	**Mean**	**SD**	**FAI**	**SAS-SV**	**PHQ-2**	**GAD-2**
FAI	0.83	30.14	21.89	1.00	–	–	–
SAS-SV	0.92	40.82	10.13	0.31^**^	1.00	–	–
PHQ-2	0.73	2.32	1.44	0.39^**^	0.30^**^	1.00	–
GAD-2	0.81	2.18	1.53	0.41^**^	0.32^**^	0.65^**^	1.00

^**^p < 0.01.

α, Cronbach's alpha coefficient; FAI, Fonseca Anamnestic Index; SAS-SV, Smartphone Addiction Scale—Short Version; PHQ-2, Patient Health Questionnaire-2; GAD-2, Generalized Anxiety Disorder-2; SD, standard deviation.

In addition, the scores of each item of FAI in the PSU subgroup were significantly higher than those in the non-PSU subgroup (*p* < 0.05). The mean score difference of neck pain (2.55) was the largest, followed by malocclusion (1.95), emotional tension (1.86) and TMJ pain (1.61) (Table 3).

**Table 3:** Mean scores and difference with PSU scores of each item.

**FAI**	**SFAI**	**Item**	**Non-PSU**	**PSU**	**Mean score difference**	***p***
Q1	Q1	Opening difficulty	1.36	2.19	0.83	0.04
Q2	Q2	Side movement difficulty	0.91	2.10	1.19	< 0.01
Q3	Q3	Muscle pain	1.62	2.79	1.17	< 0.01
Q4		Headache	2.27	3.55	1.28	< 0.01
Q5		Neck pain	2.79	5.34	2.55	< 0.01
Q6	Q4	TMJ pain	1.23	2.84	1.61	< 0.01
Q7	Q5	TMJ noise	2.01	3.58	1.57	< 0.01
Q8		Parafunction	1.10	2.35	1.25	< 0.01
Q9		Malocclusion	1.88	3.83	1.95	< 0.01
Q10		Emotional tension	2.21	4.07	1.86	< 0.01

FAI, Fonseca Anamnestic Index; SFAI, short-form Fonseca Anamnestic Index; PSU, problematic smartphone use; TMJ, temporomandibular joints.

Univariate analysis indicated that age, depression, anxiety and PSU were risk factors for the presence of TMD. The multiple logistic regression analysis involved SAS-SV and FAI scores as dichotomous variables according to corresponding cutoffs. Age, depression and anxiety were involved as confounders. After adjusting for potential confounders, subjects with PSU were more likely to report TMD than those without PSU (OR = 1.77, 95% CI 1.04–3.06). As for PT, depression, anxiety and PSU were risk factors for it. And compared to people without PSU, people with PSU had a higher prevalence of PT (OR = 1.81, 95% CI 1.08–3.04) after adjusting for depression and anxiety. In addition, the risk factors for IT were only depression. After adjusting for age, depression and anxiety, PSU had no association with IT (*p* = 0.08) (Table 4).

**Table 4:** Univariate and multivariate logistic regression analysis of variables associated with the presence of TMDs, PT, and IT (*N*=470).

**Variate**	**TMD**		**PT**		**IT**	
	**OR (95%CI)**		**OR (95%CI)**		**OR (95%CI)**	
	**Crude**	**Adjusted**	**Crude**	**Adjusted**	**Crude**	**Adjusted**
**Age**	1.17 (1.08, 1.27)	1.17 (1.07, 1.27)	1.04 (0.97, 1.10)		1.08 (1.01, 1.15)	1.06 (0.99, 1.13)
*p*	< 0.01	< 0.01	0.28		0.03	0.11
**Gender**						
Male	Reference		Reference		Reference	
Female	1.29 (0.87, 1.93)		0.94 (0.64, 1.38)		0.95 (0.65, 1.40)	
*p*	0.20		0.74		0.80	
**Income**						
< 3,000	Reference		Reference		Reference	
>3,000	0.98 (0.61, 1.57)		0.82 (0.52, 1.29)		1.26 (0.81, 1.98)	
*p*	0.95		0.40		0.30	
**Depression**						
No	Reference	Reference	Reference	Reference	Reference	Reference
Yes	4.17 (2.63, 6.61)	2.51 (1.48, 4.24)	2.50 (1.68, 3.71)	1.63 (1.03, 2.59)	2.79 (1.87, 4.16)	2.20 (1.37, 3.52)
*p*	< 0.01	< 0.01	< 0.01	0.04	< 0.01	< 0.01
**Anxiety**						
No	Reference	Reference	Reference	Reference	Reference	Reference
Yes	4.30 (2.62, 7.07)	2.46 (1.40, 4.33)	2.76 (1.82, 4.20)	1.93 (1.19, 3.14)	2.28 (1.51, 3.34)	1.39 (0.86, 2.26)
*p*	< 0.01	< 0.01	< 0.01	< 0.01	< 0.01	0.18
**PSU**						
No	Reference	Reference	Reference	Reference	Reference	Reference
Yes	2.69 (1.63, 4.42)	1.77 (1.04, 3.06)	2.35 (1.43, 3.87)	1.81 (1.08, 3.04)	2.09 (1.28, 3.43)	1.59 (0.95, 2.68)
*p*	< 0.01	0.03	< 0.01	0.03	< 0.01	0.08

PSU, problematic smartphone use; TMD, temporomandibular disorder; PT, pain-related temporomandibular disorder; IT, intra-articular temporomandibular disorder; OR, odds ratio; CI, confidence interval.

Then a stratified analysis of the results was performed. The risk of TMD in people with PSU was higher than that in people without PSU (OR = 1.82–4.35), although the difference was not statistically significant in the depression subgroup and anxiety subgroup. The odd ratio for TMD was the highest among the group with monthly incomes of < 3,000 (OR = 4.35, 95% CI 1.42–13.33). Interaction analysis revealed that PSU was associated with TMD in all subgroups. As for PT, people with PSU had a higher prevalence of PT in comparison to people without PSU (OR = 1.47–11.53) except for some groups in which the difference was not statistically significant. The association between PSU and PT was universal in all subgroups but people with and without anxiety. In addition, in most subgroups there was no significantly statistical difference in the IT prevalence between people with and without PSU, which was consistent with the results of multivariate logistic regression analysis (Table 5).

**Table 5:** Association between PSU and TMD, PT and IT according to baseline characteristics.

**Subgroup**	**TMD**			**PT**			**IT**		
	**OR (95%CI)**	***p***	***p*** **for interaction**	**OR (95%CI)**	***p***	***p*** **for interaction**	**OR (95%CI)**	***p***	***p*** **for interaction**
Gender			0.81			0.67			0.41
Male	2.84 (1.30, 6.22)	< 0.01		2.72 (1.24, 5.95)	0.01		2.72 (1.24, 5.95)	0.01	
Female	2.51 (1.31, 5.00)	< 0.01		2.17 (1.14, 4.16)	0.02		1.78 (0.93, 3.38)	0.08	
Income			0.34			0.56			0.94
< 3,000	4.35 (1.42, 13.33)	0.01		1.77 (0.60, 5.19)	0.30		2.18 (0.72, 6.55)	0.17	
≥3,000	2.37 (1.36, 4.15)	< 0.01		2.55 (1.45, 4.48)	< 0.01		2.08 (1.19, 3.62)	< 0.01	
Depression			0.86			0.21			0.29
Yes	1.82 (0.46, 7.18)	0.39		3.83 (1.16, 12.67)	0.03		0.88 (0.23, 3.41)	0.86	
No	2.07 (1.18, 3.63)	0.01		1.65 (0.94, 2.90)	0.08		1.88 (0.23, 3.41)	0.03	
Anxiety			0.56			0.01			0.82
Yes	3.15 (0.73, 13.61)	0.13		11.53 (2.29, 58.12)	< 0.01		2.05 (0.52, 8.01)	0.30	
No	1.96 (1.14, 3.37)	0.02		1.47 (0.86, 2.53)	0.16		1.72 (1.00, 2.97)	0.05	

PSU, problematic smartphone use; TMD, temporomandibular disorder; PT, pain-related temporomandibular disorder; IT, intra-articular temporomandibular disorder; OR, odds ratio; CI, confidence interval.

## Discussion

This study focused on the relationship between PSU, specific TMD, and psychological distress among young adults. Our study showed that TMD was associated with age, depression, anxiety, and PSU, and the subjects with PSU showed a higher risk of TMD than those without PSU. Considering the significant association between TMD and PSU, the first null hypothesis was rejected. Moreover, PSU also increases the presence of PT, therefore the second null hypothesis was duly discarded. However, there was no association between IT and PSU when the ages and psychological problems were separated, so the third null hypothesis was accepted.

The prevalence of TMD symptoms was 66.4%, which was consistent with that of Schaidt et al. (10) who reported a 64.1% prevalence among college students. Subjects with psychological problems had a significantly higher risk of TMD than those without psychological problems. A previous study also indicated that depression and anxiety had a significant effect on TMD symptoms (27).

There was no significant difference in the prevalence of TMD between different genders. However, the prevalence of TMD in females was significantly higher than those in males in both PSU group and non-PSU group, which was similar to the founding by Almasan et al. (29). The higher prevalence of TMD in women may be attributed to many factors, such as hormones, pain sensitivity, psychological factors as well as sociocultural factors (30). Estrogen may cause joint tissue flaccid and increase the risk of TMD (31). Additionally, no difference was observed in the prevalence of TMD between income subgroups, in agreement with the study of Martins et al. (32). One possible explanation is that college students have many sources of income, like scholarships and part-time jobs, and don't worry about family income. So significant difference in the prevalence of TMD between different incomes was not observed.

In the diagnosis of TMD, DC/TMD is a validated and accurate method (16). But DC/TMD is not suitable for screening for TMD in large populations, because it is time-consuming and difficult to apply. FAI is a commonly used and popular screening method for TMD, owing to its low cost, simplicity, and convenience (23). Previous studies showed that the FAI has high sensitivity and specificity for detecting the presence of TMD (33). Zhang et al. (24) reported that the sensitivity and the specificity of the FAI Chinese version were 95.9% and 71.9%, respectively. As a fast, easy, and inexpensive instrument, it is widely applied in clinical and in epidemiological surveys. Hence, we choose FAI instead of DC/TMD in our study.

The result showed that the prevalence of PSU (83.6%) was higher than that of Chinese medical students (49.7%) from an online survey on July 2020 (34). Consistent with our results, PSU prevalence for young adults was reported to be 71.9% in Saudi Arabia (35). The high prevalence of PSU among university students could be attributed to several reasons. Firstly, due to the COVID-19 pandemic, many young adults had to use virtual platforms to complete their academic and work tasks, which might exacerbate smartphone use (36). In addition, subjects with psychological problems had a significantly higher risk of PSU. A study proved that smartphone addiction was related to many psychological disorders, such as depression and anxiety (37). University students often face peer pressure and many other situations which might increase the risk of depression and anxiety. After a long day at study and work, young adults might use their smartphones for getting pleasure and gratification to relax themselves (38).

Recently, researchers found a statistically significant positive correlation between smartphone addiction and cervical pain of students in Brazil (39). It had been observed that various aspects of smartphone use increased the risks of bruxism and TMD (40). Previous studies indicated that all smartphone tasks would lead to a significant increase in trunk inclination (41, 42), which possibly affects head flexion. In the study, the FAI score was moderately statistical correlated with the SAS-SV score (*r* = 0.31), and the young adults with PSU were 1.77 times more likely to present TMD symptoms than those without PSU. All ten symptoms of TMD were more serious in individuals with PSU than those without PSU, and the difference in mean score of neck pain (2.55) was the largest, which means the correlation between neck pain and PSU was strongest. This relationship between neck pain and PSU might have something to do with the posture when using smartphones. A previous study had shown that head and neck positioning was altered when using smartphones, and that forward head posture may result in increased mechanical loading of the joints, neck, and cervical spine ligaments (43). Bae and Park (44) found the muscle activation level of the trapezius muscle would decrease after using smartphones, which might cause muscle fatigue. While using smartphones, young adults usually keep the head and neck flexed, which consequently changes the electrical activity of both the cervical and masticatory muscles. Because of the interdependence of these regions, injury or disease in one area could induce dysfunction and pain in others. The above studies supported our findings that PSU is related to TMD symptoms.

Numerous laboratory-based electromyography (EMG) researches reported that people with TMJ pain had slightly increased muscle activity. Previous study showed that people with TMD had more frequent muscle activity (45). But Hilal Başak et al. (46) found that smartphone overuse behaviors didn't affect the endurance of deep cervical flexors (DCF) muscles through SAS-SV and assessment of endurance of DCF muscles. The difference might be attributed to the differences in measurement methods. This study suggested that there might be other pathways of association between PSU and TMD. And the pathogenesis of TMD is various and complex. PSU and TMD might be linked through other mechanisms, such as psychological problems. Psychological distress was also a possible agent for the relationship between PSU and TMD symptoms. The correlations between PSU and psychological problems were observed in this study (*r* = 0.30–0.32). Our results were supported by an early longitudinal study reporting that PSU increased psychological distress among Hong Kong university students (47). Furthermore, a cohort study exhibited that depression and stress increased the risk of TMD (48). The literature which described the association between pain and depression confirmed our research (49). Some studies reported psychological factors could predispose people to TMD-pain, precipitate the pain or prolong the pain (11). We hypothesized that PSU might increase the risk of TMD-related symptoms through psychological factors. Therefore, future studies are required to demonstrate the effect of psychological problems on the relationship between PSU and TMD.

Subjects who were positive for PSU had a significantly higher risk to present PT rather than IT than those who were PSU-free. Interestingly, a recent study reported that patients with PT were more likely to experience neck hypomobility and muscle impairment than patients with IT (50). Considering that pain is usually in connection with motor impairment (51), the finding of the present study might be explained that the stimulation from neck and masticatory structures converge into the same neuroanatomical structure, the trigeminocervical complex. Thus, the poor neck posture when using smartphones may lead to pain in the masticatory system.

Overall, TMD might disturb the individuals' daily life, with harm to physical and psychological health and social performance, which not only affects the nutrition intake, but also increases the risk of respiratory disorders and vertigo (52–54). Currently, TMD could be prevented and treated by changing bad habits (55). This study suggests doctor and dentist educate patients to use smartphones less for prevented TMD or relieve symptoms. Additionally, TMD should be addressed promptly, because chronic pain with psychological problems and somatization becomes more difficult to treat. This finding might aid the establishment of treatment at an adequate time, avoiding setting the irreversible lesions.

This study had some limitations. Firstly, the cross-sectional design did not provide evidence of causal and temporal relations between PSU and TMD. Longitudinal designs are needed to reveal true causal linkages. Beside that, although the standard measurements were used to evaluate the PSU, the duration and purpose of smartphone use were not collected, which might overestimate the true prevalence of PSU. In addition, the enrolled sample only involved adults and excluded adolescents, which might reduce the applicability of the study. The inclusion of adolescent and pediatric subjects should be considered for future work. Finally, this information was obtained from a self-assessment questionnaire and might thus be susceptible to anamnestic bias. In the future, both FAI and DC/TMD would be employed to screen and diagnose TMD.

## Conclusion

PSU and TMD are common in young adults (83.6 and 66.4%). The univariate and multivariate regression analysis showed that age, depression, anxiety and PSU were risk factors for TMD. In the stratified analysis, the TMD prevalence of participants with PSU was higher than those of people without PSU in all subgroups. People with PSU had significantly higher risk of PT rather than IT than those without PSU. Our results suggest it is necessary to pay attention to the smartphone use of TMD patients in the diagnosis and treatment of TMD. Young adults could reduce the risk of TMD by spending less time on smartphones. Further studies should be conducted by using methods standardized with a larger sample group for the final decision.

## Data availability statement

The original contributions presented in the study are included in the article/supplementary material, further inquiries can be directed to the corresponding authors.

## Ethics statement

The studies involving human participants were reviewed and approved by Ethics Committee of West China School of Stomatology of Sichuan University. The patients/participants provided their written informed consent to participate in this study.

## Author contributions

XX designed and led the overall study. Y-PP and H-CL contributed to data collection, organization and analysis, sample selection, and wrote the first version of the manuscript. J-WZ, X-LG, C-QX, and YY collected data and revised the manuscript. All authors read and approved the final manuscript.
